# Fiberless multicolor neural optoelectrode for in vivo circuit analysis

**DOI:** 10.1038/srep30961

**Published:** 2016-08-03

**Authors:** Komal Kampasi, Eran Stark, John Seymour, Kyounghwan Na, Herbert G. Winful, György Buzsáki, Kensall D. Wise, Euisik Yoon

**Affiliations:** 1 Department of Biomedical Engineering, University of Michigan, Ann Arbor, MI 48109, USA; 2 NYU Neuroscience Institute, School of Medicine, East River Science Park, Alexandria Center, 450 East 29th St, 9th Floor, New York, NY 10016, USA; 3 Department of Physiology and Pharmacology, Sackler Faculty of Medicine, Tel Aviv University, 69978 Tel Aviv, Israel; 4 Sagol School of Neuroscience, Tel Aviv University, 69978 Tel Aviv, Israel; 5 Department of Electrical Engineering and Computer Science, University of Michigan, Ann Arbor, MI 48109, USA

## Abstract

Maximizing the potential of optogenetic approaches in deep brain structures of intact animals requires optical manipulation of neurons at high spatial and temporal resolutions, while simultaneously recording electrical data from those neurons. Here, we present the first fiber-less optoelectrode with a monolithically integrated optical waveguide mixer that can deliver multicolor light at a common waveguide port to achieve multicolor modulation of the same neuronal population *in vivo*. We demonstrate successful device implementation by achieving efficient coupling between a side-emitting injection laser diode (ILD) and a dielectric optical waveguide mixer via a gradient-index (GRIN) lens. The use of GRIN lenses attains several design features, including high optical coupling and thermal isolation between ILDs and waveguides. We validated the packaged devices in the intact brain of anesthetized mice co-expressing Channelrhodopsin-2 and Archaerhodopsin in pyramidal cells in the hippocampal CA1 region, achieving high quality recording, activation and silencing of the exact same neurons in a given local region. This fully-integrated approach demonstrates the spatial precision and scalability needed to enable independent activation and silencing of the same or different groups of neurons in dense brain regions while simultaneously recording from them, thus considerably advancing the capabilities of currently available optogenetic toolsets.

Neural circuits in the brain are intricately woven to govern complex signal pathways responsible for thought, memory, emotion and behavior. The dynamic relations between neural pathways can be studied by manipulating local populations of neurons *in vivo* in a controlled manner. The emerging field of optogenetics is allowing scientists to control and map brain circuits with cell-type specificity at high spatial and temporal precision^1^,^2^,^3^. Optogenetics is based on the genetic transfection of specific cell types to express photosensitive proteins (called opsins), which can then be controlled by wavelength-specific light. Opsins display a wide range of spectral sensitivity and can be used to activate (depolarize) or silence (hyperpolarize) the targeted neurons, with the aim of understanding neural computation^4^,^5^,^6^. For example, Channelrhodopsin-2 (ChR2), responds to ∼470 nm light and depolarizes the targeted cells^1^,^7^. Other opsins like Archaerhodopsin (Arch)^8^,^9^ and Halorhodopsins (NpHr)^10^,^11^, when illuminated with ~590 nm light, induce hyperpolarization. Multiple opsins can be expressed in the same cell^10^,^12^ or in different cell types^6^,^13^,^14^ to specifically target and manipulate local circuit elements.

Although the number of novel opsins and solutions for cell-type specific expression is increasing^15^,^16^,^17^, technology that can reliably deliver spatially-confined multicolor light and simultaneously record from small neuronal groups in behaving animals is not yet available. Combining precise optogenetic control with reliable electrophysiological readout is a technological challenge, and is essential for understanding neural circuit dynamics. Early solutions to deliver light to deep brain structures while simultaneously recording from neurons involved manual assembly of commercially available optical and recording components, resulting in bulky device assemblies^18^,^19^. Moreover, stimulation through relatively large light sources placed on the surface of the brain^20^ or large fibers (core diameter, ∼200 μm) placed in the brain parenchyma^21^,^22^, inevitably activates many un-monitored neurons. Recently, we reported the first monolithically integrated optical waveguide in a multi-electrode silicon probe, delivering light in the proximity of precisely-defined recording sites^23^. Spatially confined light (473 nm) from a DPSS (diode-pumped solid-state) laser was delivered through a fiber to the waveguide on the neural probe. However, this approach is not scalable since applying light at multiple brain sites independently would require multiple external fibered-light sources, which would constrain animal movement. Independently, we demonstrated a complete multi-site/multi-color optical stimulation and electrical recording system using light sources (LED chips and/or laser diode can mounts) attached to commercial silicon recording probes and/or wire tetrodes^24^. While this approach offers multicolor light stimulation, light delivery cannot be achieved at a common site and the assembly procedure is labor-intensive and prone to inaccuracies. Another approach involved the direct assembly of laser chips on a silicon probe back-end^25^; that effort provided a monochromatic (650 nm) optogenetic tool with integrated SU-8 waveguides, yet *in vivo* testing was not reported and device heating was not addressed.

In this report, we describe the design and in vivo use of a novel fiberless multicolor optoelectrode. The key design element is the coupling of compact ILDs to a monolithic oxynitride optical mixer waveguide on a silicon probe through GRIN lenses^26^. Figure 1 shows schematic of a GRIN-based optoelectrode assembly and Fig. 2 shows the working prototype of the device. The dielectric mixer enables multicolor stimulation at a scalable common waveguide port (7 × 30 μm) (Supplementary Movie S1), a novel feature that allows addressing neuroscience questions requiring, for instance, independent activation (with 405 nm light) and silencing (with 635 nm light) of the cells within a given locality. The monolithically integrated iridium electrode sites have a 20 μm pitch, facilitating high-density recordings from dense brain regions such as the CA1 pyramidal layer of the mouse hippocampus. The integrated GRIN lens mounted on a customized “ILD-GRIN jig” offers several advantages over alternative, conventional approaches for compact optoelectronic designs. It collimates and focuses the in-coupled divergent laser beam. The flat GRIN ends facilitate efficient butt-coupling and lenses can be designed with a diameter as small as 250 μm. This simple geometry in a miniature package allows efficient and compact optical coupling and assembly for microscale optoelectronic devices. The wide misalignment tolerance range offered by the GRIN lens maintains reproducible assembly and high yield during production. Finally, GRIN lenses provide good thermal isolation between the ILDs and the silicon probe, minimizing tissue heating. We tested the resulting optoelectrode device by recording, activating, and silencing pyramidal cells in the hippocampal CA1 region of intact mice. The design is scalable and will enable performing combinatorial experiments at deep brain regions. Such experiments may involve (1) independent activation and silencing of a single cell type; (2) activation of one source of inputs to a given cell while silencing a second source; and (3) independent activation (or silencing) of two spatially intermingled cell types.

## Results

### Optical design

The angular diffraction exhibited by lasers can cause considerable optical loss when coupling light into small symmetrical elements such as optical waveguides. To optimize coupling efficiency between a divergent laser beam and a step-graded waveguide, we implemented a collimation-focusing mechanism using a GRIN lens^27^,^28^. Since a GRIN lens has a continuous change of the refractive index (RI) within the lens material, light rays can be continuously bent within the lens until they are finally focused on a spot. GRIN-based optical coupling requires exact, design-specific spacing between the coupled components (ILD, GRIN and waveguide), which can be reliably achieved using micro-electro-mechanical systems (MEMS) fabrication. GRIN design parameters including numerical aperture (NA), working distances (L1, L2), and mechanical length (Z), were optimized to achieve the desired magnification (M < 1) for enhanced optical coupling. The design was then shared with the lens manufacturers (NSG, Japan, via Go!Foton lens distributors in Somerset, NJ, USA). Primary GRIN design equations are: RI at radius r, 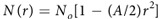
27; mechanical length, 
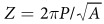
27; numerical aperture, *NA* = *n*_*o*_ sin *θ*_*a*_, where N_o_ is the RI at the lens central axis (1.65); √A is the designed index gradient constant (mm^−1^), which depends on lens material and wavelength; P is a lens pitch (fraction of a full sinusoidal period of ray path); n_o_ is the RI of surrounding medium around GRIN; and θ_a_ is the lens acceptance angle (25 degrees).

For efficient coupling of the GRIN lens to the waveguide, the latter should have an NA equal or higher than the former. Then, all incoming rays from the GRIN lens can be efficiently collected by the waveguide if aligned perfectly, and the only loss occurring at the coupling interface are reflection (Fresnel) losses. Fresnel losses are given by 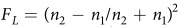, where n_1_ and n_2_ are the RIs of the first and second media, respectively. In the current implementation, the waveguide NA is 0.4228 (designed to closely match the NA of the GRIN lens, 0.4226) using 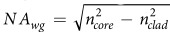, where n_core_ is the RI of the waveguide core (silicon oxynitride, 1.52) and n_clad_ is the RI of the waveguide cladding (silicon dioxide, 1.46). Using the Fresnel equation, reflective losses were calculated as 0.462 dB at the ILD-GRIN junction (assuming an intermediate medium with RI = 1.56) and 0.463 dB at the GRIN-waveguide junction, yielding a total coupling loss of 0.925 dB (i.e. >80% total coupling efficiency from ILD to waveguide).

Our waveguide design is based upon parametric ray tracing models (Zemax LLC, Kirkland, WA, USA) shown in Fig. 3a. We chose a full-pitch (P = 1) GRIN lens of high NA which gives a focused beam at the GRIN output as the beam travels exactly one full cycle of a sinusoidal period in that distance, achieving beam focusing on the other end (Fig. 3a, ***inset***). The focused beam enters the tapered waveguide mixer arms and then into the straight waveguide. Due to optical mode distortion, radiation losses occur in the waveguide bends. These losses can be minimized by designing the bend with a large radius of curvature. However, large curvature comes at the cost of a longer light path, resulting in higher transmission losses and larger device size, which is often limited by the maximum tolerance of pitch for micro-optical assemblies (in our case, limited by the diameter of the GRIN lenses). Due to this tradeoff, we designed the mixer with a maximum bend radius of 2.32 mm while maintaining a minimum pitch between GRIN lenses; and achieved simulated radiation loss within 1 dB^29^,^30^. Other than coupling and radiation loss, light rays also suffer from as propagation loss, which is attenuation in the form of scattering and absorption as they travel through the waveguide. The total optical loss of the system, L_T_, is sum of all three loss-types:
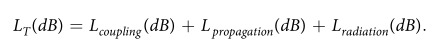


The waveguide aperture on the neural shank was positioned 55 μm away from the first recording site to minimize damage to the recorded neurons^21^,^24^,^31^. Since the recording sites span 140 μm, opsin activation thresholds must be crossed at a distance of ∼200 μm from the tip of the of 7 μm × 30 μm waveguide. The design values used were: 405 nm light, intensity of 2 mW/mm^2^ for ChR2^32^,^33^; and 635 nm, intensity of 7 mW/mm^2^ for Halo/Arch^12^,^34^. Considering waveguide geometric losses and tissue scattering losses through brain tissue for each wavelength^24^,^33^, the required light intensity is achieved at a distance of 200 μm from the waveguide if the output power (intensity) at the waveguide tip exceeds 100 μW (476 mW/mm^2^) for 405 nm and 200 μW (952 mW/mm^2^) for 635 nm (Fig. 3b).

### Thermal design

Although there is no established temperature threshold for safe operation of neural probes when implanted in brain tissue^35^, temperature can affect neuronal activity on cellular and population level in various manners^36^,^37^,^38^. Therefore, we loosely define the design threshold as 1 °C temperature rise from the baseline tissue temperature of 37 °C for a conservative thermal model analysis^39^.

Optical power above 200 μW must be emitted at the 7 × 30 μm waveguide tip to achieve optogenetic activation in tissue as far as 200 μm away (Fig. 3b). Due to the high optical efficiency provided by the GRIN-based design, this can be achieved using low-power ILDs and driving them just above their stimulated emission threshold, at an input electrical power of ∼80 mW. For conservative modeling, we assumed all electrical input power is dissipated as heat. We used a computerized heat transfer model (COMSOL Multiphysics, Burlington, MA, USA) to simulate the temperature rise of the electro-optical components and the tissue around the optoelectrode. The simulation results (Fig. 4a,c) indicate that both ILDs can be driven continuously for 190 s just above their threshold current (200 ms pulse width, 10% duty cycle), which is more than adequate for most optogenetic circuit-analysis applications^24^. The maximal temperature of the ILDs themselves (after 190 s at 10% duty cycle) is 50.4 °C, which is within the specified safe operational temperature^40^. In an extreme case, when ILDs are driven by DC current, the continuous device operation time is reduced to 45 s, with a maximal ILD temperature of 52.4 °C.

The GRIN-coupled design (Fig. 4a) prolongs device operation time more than 2-fold as compared to a conventional design, in which diodes are directly butt-coupled to the waveguides without intermediate optical lenses (Fig. 4b). This has a critical influence on the thermal budget when scaling the dual-ILD/single-shank device to multi-shank probes. As the number of diodes per device increases, the electrical power consumed, and hence the dissipated heat, increases. We simulated the temperature rise at the tissue surface for GRIN-coupled and butt-coupled designs, for 2-, 8- and 16-diode assemblies (Fig. 4c,d). The higher thermal resistance of the GRIN lenses helps manage the heat generated by the light sources without the use of active (e.g. convective or thermoelectric) coolers. Compared to the butt-coupled design where the rise in temperature is fast and oscillatory, the higher thermal constant offered by the GRIN-coupled design facilitates a slower and continuous temperature rise at the tissue, allowing future scaling in term of the numbers of shanks and diodes.

### Device fabrication

Our modular fabrication process follows Michigan probe microfabrication technology^23^,^41^ with monolithic integration of a waveguide mixer (Fig. 5a–d) and fabrication of a custom heat sink (ILD-GRIN jig) for micro-optic assembly of ILDs and GRINs (Fig. 5e–g). The two fabricated components are then assembled together on a PCB (Fig. 5h). The details of the fabrication are assembly processes are discussed in the Methods section of this article. Figure 5i shows the SEM image of the released neural probe and Fig. 5j shows the magnified SEM view of the waveguide structure fabricated on the top of the probe. The colored image of the released ILD-GRIN jig is shown in Fig. 5k (without assembled components) and Fig. 5l (with assembled components).

### Optical characterization

#### ILD efficiency

An effective diode packaging solution can help to quickly dissipate the excessive heat generated in the diode to its surroundings and enhance device reliability. We efficiently managed the heat dissipation from the ILDs to the ILD-GRIN jig (heat sink) and to the PCB. The anode of the epi-side-down bonded ILDs quickly diverted the thermal flux from the diodes to the designated heat sink. The ILD cathodes were grounded (via wirebonds and thermal conductive epoxy) to the ground plane of the PCB.

The effectiveness of an ILD assembly is evaluated from its wall-plug efficiency (or radiant flux), which is the efficiency at which the diode assembly converts input electrical power into output optical power. We measured a wall-plug efficiency of 4.48% (for 405 nm) and 5.49% (for 635 nm) for packaged ILDs (Fig. 6a).

#### System optical loss

**Figure 2:**
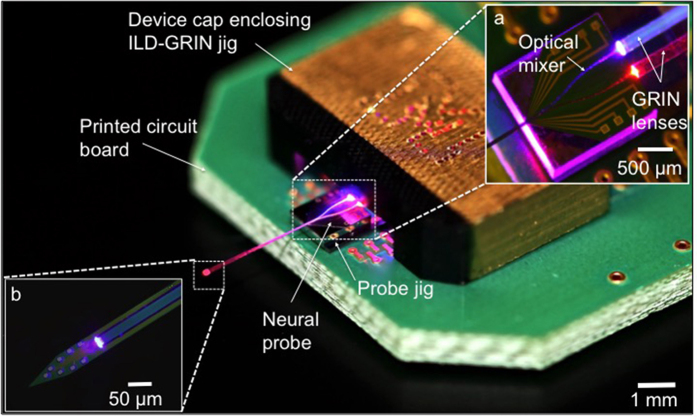
Working device prototype assembled on a PCB. Inset (**a**) shows the enlarged view of the optical mixer at the back end of the probe with GRIN lens coupling into the two arms of the waveguide mixer. Inset (**b**) shows the enlarged probe tip with color mixed light illuminating at the 30 μm x 7 μm waveguide tip. In a finished device, the device cap front is sealed with an opaque epoxy. Also, see Supplementary Movie S1.

We quantified optical losses in each part of the system separately: (1) coupling loss at the ILD-GRIN and GRIN-waveguide junctions; (2) radiation loss in the bends and corners of the optical mixer; and (3) propagation losses through the waveguide. Measurement using the direct cut-back method was used to evaluate propagation loss per unit length of a straight waveguide (Fig. 6b). The observed slope of the linear fit, 0.5 dB/mm, gives the waveguide propagation loss. The y intercept (at 0 mm length) of the linear fit, 1.76 dB, gives the total coupling (including Fresnel) loss between the GRIN lens and waveguide, including back reflection at the tip of the waveguide. The coupling loss from ILD to GRIN output was separately estimated as 0.5 ± 0.1 dB (mean ± s.d., N = 5) by comparing optical power at the ILD (635 nm) and ILD-GRIN outputs. Radiation losses from straight channel waveguides are generally negligible for well-confined modes but may increase in waveguide bends. Our mixer geometry has two bends per light path, and we measured radiation losses of 1.4 ± 0.3 dB (mean ± s.d., N = 5) when coupled to 635 nm ILD source. The summed losses of all sources measured for 635 nm light during bench testing was 7.18 ± 0.22 dB for the complete waveguide length (7.04 mm). However, the optical loss measured for packaged devices (Fig. 2) was 11.7 ± 1.1 dB and 9.9 ± 0.7 dB (mean ± s.d., N = 5) for 405 nm and 635 nm, respectively, which is ∼27% higher than estimated values from experimental devices. This may be mainly due to misalignment in the micro assembly of optical components on a common substrate PCB in the packaged devices. Nevertheless, the experimental range of total optical loss of 9.2–12.8 dB (with 5.2–12% coupling efficiency) is, to the best of our knowledge, the highest reported to date for diode-coupled optoelectrodes. Previous work on ILD-coupled SU-8 waveguides reported ∼30 dB loss with only one integrated wavelength (650 nm)^25^. Other work reported a multicolor diode assembly with 26 dB loss for blue (465 nm) LEDs and 13 dB loss for red (639 nm) ILDs coupled into 50 μm (core diameter) fibers^24^. Other efforts reported comparable optical losses for a single wavelength, yet with high-power DPSS (diode-pumped solid-state)-based systems^23^,^42^. Our packaged device yielded, when coupled to a 6 mW ILD, an average output intensity of 1928 mW/mm^2^ (405 μW output power) for 405 nm and 2905 mW/mm^2^ (610 μW) for 635 nm at the waveguide tip.

#### Optical misalignment tolerance analysis

Most alignment errors in micro-optics come from component mismatch and assembly misalignment. Using ray tracing modeling, we investigated the effect of misalignment tolerances on the GRIN-based optoelectrode; we then compared the simulation results with bench tests (Fig. 7a). Among all optical coupling interfaces, the ILD-GRIN coupling junction is the most tolerant. Large misalignment margins were obtained when the GRIN lens was misaligned in X and Y (lateral symmetrical GRIN axes) or Z (longitudinal) axes with respect to the ILD (Fig. 7b,c), allowing up to ±115 μm lateral and 20 μm longitudinal misalignment with <10% relative optical loss. This gives a huge error margin in microfabrication when defining GRIN slots. Figure 7d–f shows normalized coupling when the mixer waveguide (WG) is misaligned with respect to the ILD-GRIN assembly in the X, Y and Z-axes, respectively. Here, the axis most sensitive to misalignment is Y (Fig. 7e), where tolerance is dictated by the height of the WG core. In order to accurately control the vertical GRIN-WG alignment, the emission point of an ILD should be aligned to the center of a WG cross-section by selecting the precise height of the probe jig. Since this jig is easily replaceable, the GRIN lens can be reliably and reproducibly positioned between the ILD and WG. Even if the GRIN lens is misaligned at this step, relatively large normalized output power of 90%, 95% and 70% can be achieved within a tolerance of ±25 μm in X and Y axes and 10 μm in Z-axis, respectively (Fig. 7g–i). These alignment margins can be easily accomplished during the assembly process.

### Electrical characterization

Impedance and electrical noise of recording channels were measured in phosphate buffered saline (PBS, 0.1 M, Fisher Scientific, Hampton, New Hampshire, USA) with an RHD2164 amplifier board connected to an RHD2000 Evaluation System (Intan technologies, Los Angeles, CA, USA). The average impedance of recording sites (140 μm^2^) was 410 ± 30 kΩ with 68 ± 2° phase at 1 kHz (mean ± s.d., N = 3 devices, 8 sites each), which is sufficiently low to record neural signals with high signal-to-noise ratio. The average baseline noise picked up by the recording channels in absence of light stimulation was 8 μV peak-to-peak.

### *In vivo* electrophysiological results

We inserted an 8-site dual-ILD silicon probe into the CA1 pyramidal cell layer of urethane-anesthetized mice. Spontaneous neural activity, including high-frequency ripple oscillations^43^ and multi-neuronal spiking (Fig. 8a, **top**), was observed on all 8 channels. When trains of 405 nm light pulses (50 ms, 1 pulse/s, 10 pulses/train; 30 mA, 100 μW at the waveguide tip) were applied, two features were evident (Fig. 8b, middle). First, the recorded pyramidal cells (PYR) increased their spiking probability, consistent with ChR2 expression driven by the CaMKII-Cre driver in these animals. Second, the local field potential (LFP) exhibited stimulus-locked transient (onset and offset) and sustained artifacts. These artifacts were comprised of fast transients and DC offset with an asymptotic attenuation, features which are consistent with capacitive effects. When the trains of 635 nm light pulses (200 ms, 1 pulse/s, 10 pulses/train; 40 mA, 370 μW at the waveguide tip) were applied through the same waveguide without moving the probe, the same cells reduced their spiking rate (Fig. 8a, bottom). The LFP exhibited similar stimulus-locked transient (onset and offset) and DC artifacts. Similar artifacts were observed in a wild-type mouse that did not express any opsins and during sham recordings in PBS (data not shown).

We quantified the cell-specific effect of light on a group of PYR (n = 19) recorded simultaneously from CA1 (Fig. 8b, inset shows the relative location of PYR and interneuron [INT] somata). Each cell was assessed for spike rate during the 405 nm light pulse, compared to baseline spiking rate (in the lack of any light). Most (11/19; 58%) of the cells increased their spike rate (p < 0.05, Poisson test), with a median gain (spike rate during light divided by baseline rate) of 15.1 and a median latency of 15 ms (see also Fig. 8b, left). Using the same approach, the same cells were also assessed for spike modulation during 635 nm light: 4/19 cells (21%) exhibited a consistent rate decrease (p < 0.05, Poisson test), with a median gain of 0.11 and a median latency of 54 ms (see also Fig. 8b, right). One PYR, the one closest to the waveguide (estimated distance from waveguide tip to soma, 75 μm), exhibited both consistent rate increase and rate decrease (p < 0.001 for both; Fig. 8c).

## Discussion

Optogenetic devices that use fibered light delivery from a bench-top source constrain free animal movement, whereas LED-coupled systems yield poor coupling efficiency because of their Lambertian light distribution profile. ILDs offer an attractive solution for optoelectrode design since they are highly compact, provide a directional beam with a wide power range, and are increasingly available in many wavelengths. However, commercial ILD packages are too large to be integrated into high-density micro-scale devices, whereas unpackaged ILD chips have divergent output beams and are also easily damaged by electrostatic pickup or excessive heat. Here, these issues were addressed by incorporating unpackaged ILD chips in a fiberless, lightweight micro-fabricated module that enabled precise assembly of optical components and facilitated protecting electrical and thermal components at the device backend. With the use of GRIN lenses as the optical coupling medium, we were able to achieve a total optical efficiency range of 5.2–12% for the assembled working prototypes (the highest reported efficiency for diode-coupled optoelectrodes to date) while facilitating thermal dissipation at device backend and providing adequate thermal insulation to the tissue.

Muti-opsin optogenetic studies require careful selection of opsins and matching light wavelengths during device design. We chose violet over blue light (usually used for ChR2 activation) because of the low sensitivity of Arch (activated here with 635 nm light), facilitating multi-opsin experimental preparations. ChR2 sensitivity to violet light (405 nm) is lower than to blue light^32^, so approximately twice the power is required when violet light is used with ChR2. Some optogenetic applications may require even higher power levels to illuminate larger populations of neurons^20^,^33^. Given the modularity of our approach, such applications can be accommodated by assembling higher power laser sources (>50 mW) on a larger heat sink, keeping other design aspects exactly the same.

The electrophysiological data indicated three clear results. First, the existence of a 9 μm high waveguide over the probe surface does not hinder neuronal recordings^21^, as spontaneous activity was recorded in each and every animal. Second, the application of 100 μW of 405 nm light was sufficient to consistently drive spiking in ChR2-expressing PYR with somata up to about 190 μm from the waveguide tip, despite the small cross-section of the waveguide core (7 × 30 μm). Although large waveguide cores can transmit more light, small cores help to confine light, resulting in higher light intensity for a given input power. Similar results were observed in CA1 of a mouse expressing ChR2 specifically in PV-cells (3/3 simultaneously-recorded INT consistently driven by 50 ms pulses of 405 nm light, p < 0.05, Poisson test; median gain, 3.2; Supplementary Fig. 1), emphasizing the wide range of potential ILD-GRIN probe applications. Third, red light power of about 400 μW (range, 50–500 μW) – despite yielding high intensity at the waveguide tip – was only partially effective at silencing spiking of nearby neurons. This is consistent with previous observations indicating that optical silencers require higher light intensity than ChR2^12^,^24^,^34^. The activation spectrum of eArch3 is blue-shifted relative to eHalo3^34^, and in our previous work, 0.4–1.3 mW of 561 nm light was required to silence PYR and suppress ripples in mouse CA1 using diode-probes^6^. These considerations suggest that although marginally effective and potentially mitigated by the development of red-shifted silencers^44^, increased red light intensity is required for robust silencing *in vivo* with the ILD-GRIN probes.

**Figure 3:**
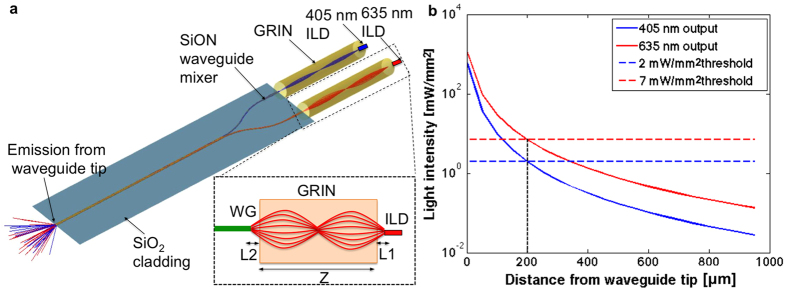
Optoelectrode optical design and light propagation. (**a**) Zemax optical model of optical mixer waveguide (7.04 mm total length) coupled to ILDs to deliver multicolor output at the single waveguide port. The model consists of two ILDs (405 nm and 635 nm) coupled to two arms (each 2 mm long) of optical mixer via respective GRIN lenses. The 405 nm (2.38 mm long) and 635 nm (2.54 mm) GRIN lenses were designed and simulated in Zemax to facilitate optimal coupling while allowing maximum misalignment tolerance between the ILDs and the waveguide. The focused beam enters the waveguide mixer arms, which taper down from a width of 50 μm to 30 μm and then converge into a 5 mm-long straight waveguide (cross-section: 30 μm × 7 μm). The schematic in the inset shows a full pitch GRIN lens collimating and focusing a divergent ILD laser beam into the waveguide mixer arm (WG). L1 and L2 denote object and image distances, respectively, that can fit well within the device fabrication and assembly precision. (**b**) Simulated light intensity curves at waveguide tip as a function of tissue depths. When output intensity at the waveguide tip is 476 mW/mm^2^ for 405 nm and 952 mW/mm^2^ for 635 nm, respectively, the tissue up to 200 μm away from the waveguide tip is illuminated at supra-threshold intensity^32^.

**Figure 4:**
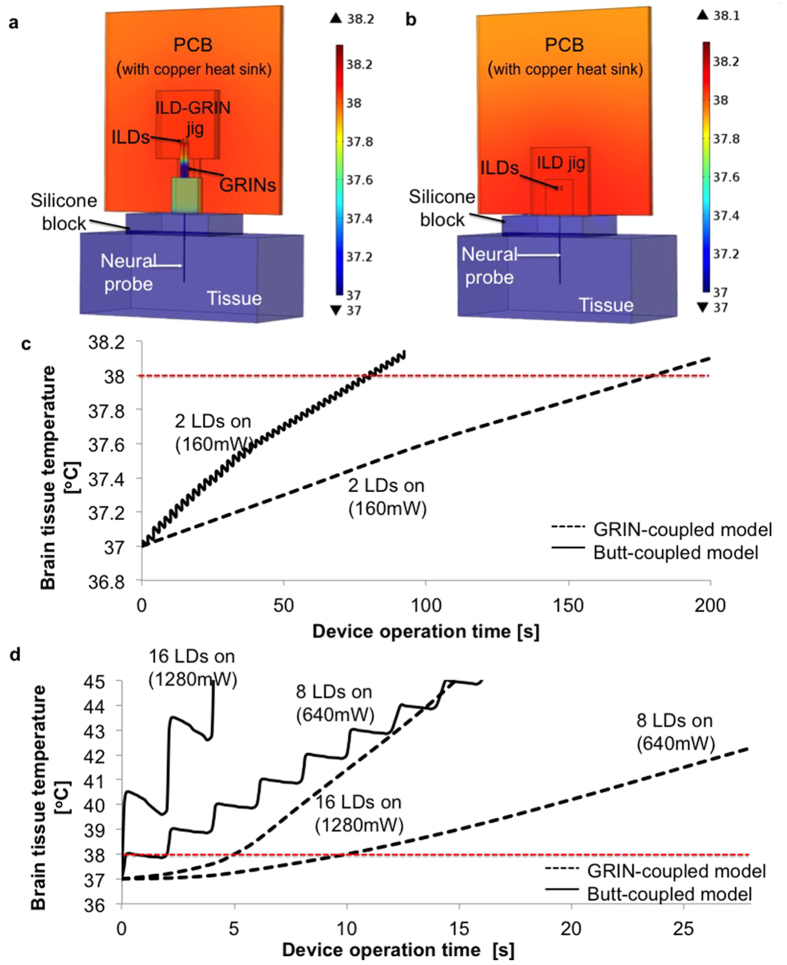
COMSOL heat transfer model results. COMSOL model for a single shank optoelectrode for (**a**) GRIN-coupled and (**b**) butt-coupled design, showing surface temperature rise of optoelectrode components and tissue surface when two ILDs are operated at 10% duty cycle power for 20 seconds. (**c**) Tissue temperature rise over time for models shown in (**a**,**b**). (**d**) Tissue temperature rise over time for multi-shank GRIN-coupled optoelectrodes compared to their design equivalent butt-coupled optoelectrodes with 8 and 16 assembled diodes. The power values on each graph line signify the total input electrical power delivered to the device, 80 mW per diode. Butt-coupled optoelecrodes show a fast and oscillatory temperature rise at their probe shanks in response to the pulsed ILD driving currents. In contrast, GRIN-coupled optoelectrodes exhibit slow and gradual temperature rise because of thermal isolation between ILDs and probe shank, offered by the thermally insulating GRIN lenses.

A limitation of the present generation of ILD-GRIN probes is the existence of stimulus-locked artifacts. While evident in many optogenetic studies^21^,^45^, the diode-probe technology^24^ has yielded artifact-free recordings^6^,^46^,^47^. The putative source of artifacts with the ILD-GRIN probes is capacitive coupling via the silicon platform jig used in device assembly (Fig. 3a), via the opening in the shield cap, and via the PCB. Modified assembly techniques, improved shield cap design, and better grounding strategies^24^ are expected to minimize these artifacts in future prototypes. Finally, while the tissue response to Michigan probes has been reported in previous studies^48^,^49^,^50^,^51^, future work in chronically-implanted animals will assess the brain-implant interaction of the ILD-GRIN probes.

## Conclusion

We have successfully fabricated, assembled, and characterized the first monolithically-integrated fiber-less ILD-GRIN coupled optoelectrode device for in vivo circuit analysis. The dielectric optical mixer waveguide integrated onto the neural probe enabled wavelength mixing at a common waveguide port, providing adequate light intensities to activate and silence local populations of same and different genetically targeted neurons. Optimal thermal packaging was achieved via efficient ILD assembly and GRIN-facilitated thermal insulation. The fully packaged optoelectrodes were tested in anesthetized mice and recorded high-quality neurophysiology, demonstrating device feasibility and potential for chronic implants. This new capability of providing multicolor stimulation at a single waveguide site facilitates independent manipulation of multiple opsins expressed in a local neural population, and is expected to play a key role in defining novel optogenetic experiments, ranging from closed-loop bi-directional control, creation of synthetic patterns to study plasticity, to control of a few to many neurons. Altogether, this will considerably advance our understanding of neural computation and unravel the local neuronal network dynamics.

## Methods

### Fabrication and assembly

**Figure 5:**
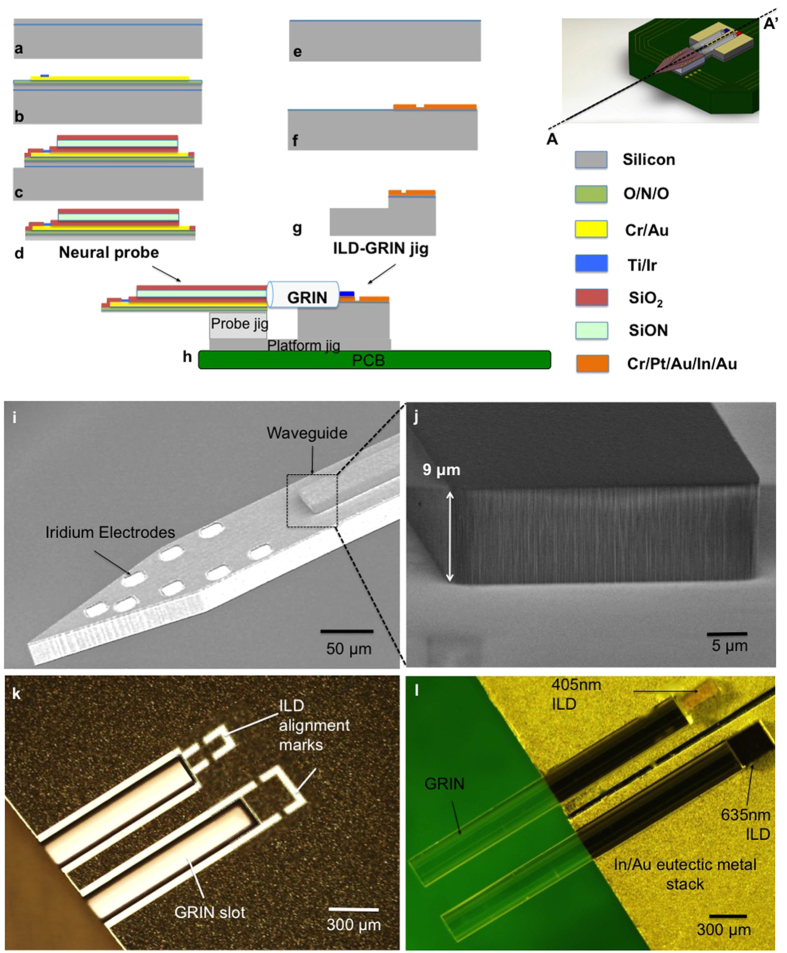
Optoelectrode fabrication and assembly on a PCB. Device fabrication along A-A’ (**a**) Begin probe fabrication on a <100> silicon-on-insulator (SOI) wafer with a 22 μm-thick silicon top layer; (**b**) deposition of LPCVD (low-pressure chemical vapor deposition) -grown silicon dioxide/silicon nitride/silicon dioxide film stack (O/N/O stack, 0.7 μm total thickness) for stress compensation and electrical insulation; followed by lift-off of Cr/Au, Cr/Au and Ti/Pt/Ir for interconnection lines, bond pads and low-impedance electrode sites, respectively; (**c**) deposition and patterning of PECVD (plasma-enhanced chemical vapor deposition) -grown waveguide films which also serve as a top insulation layer for the metals deposited in the previous steps. This is followed by dry plasma etching and wet etching (of 500 Å-thick aluminum oxide, in buffered hydrogen fluoride solution) to open contacts for bond pads and electrode sites; (**d**) front-side DRIE to define probe shank dimensions, backside thinning to release the probes from the wafer. (**e**) Begin ILD-GRIN fabrication on a <100> silicon wafer with 2 μm-thick top oxide; (**f**) deposition and pattering of metal stack of Cr/Pt/Au and In/Au for ILD flip-chipping; These metal layers were also used to define alignment marks for ILD placement (**g**) front side DRIE to etch GRIN slots followed by wafer dicing to release ILD-GRIN jigs; (**h**) Final assembly of device components on PCB. (**i**) Fabricated neural probe with monolithically-integrated dielectric waveguide and iridium electrodes in Buzsaki8 configuration. (**j**) High magnification SEM image of the dielectric waveguide tip (7 μm core with 2 μm top and 2 μm bottom cladding) fabricated on the neural probe shank. (**k**) Fabricated ILD-GRIN jig (heat sink made of silicon with eutectic metal stack) with defined ILD alignment marks. (**l**) ILD-GRIN jig with epi-side down flip-chipped 405 nm and 635 nm ILDs and assembled GRIN lenses^26^.

**Figure 6:**
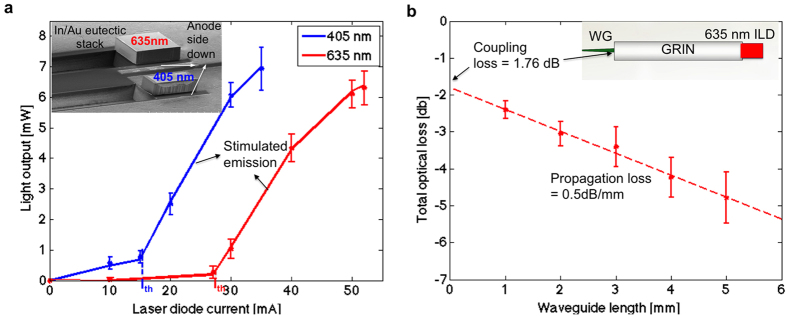
Optical characterization. (**a**) Light output-current (L–I) characteristics for epi-side down flip-chipped 405 nm and 635 nm ILDs (N = 10, data points show the mean of the collected data, and error bars represent the standard deviation). The inset shows an SEM image of the eutectic bonded ILDs. (**b**) Direct cut-back measurement for identical straight waveguide sets (N = 5, data points show the mean of the collected data, and error bars represent the standard deviation). The optical output for 5 sets of straight waveguides fabricated on the same substrate (each set consisting of five different waveguides: 5 mm, 4 mm, 3 mm, 2 mm and 1 mm long; all coupled to 635 nm LDs) was measured, and the total output loss in dB (difference between source power and measured power at waveguide output) was plotted as a function of waveguide lengths. The plotted data was used to calculate propagation loss in dB/mm and coupling loss in dB at GRIN-waveguide interface.

The neural probe fabrication (Fig. 5a–d) was started on a Silicon-on-Insulator (SOI) wafer with a 22 μm thick device layer. An LPCVD (low-pressure chemical vapor deposition) dielectric stack was grown to serve as stress compensation and electrical insulation layer. This was followed by lift-off of metal layers. PECVD (plasma-enhanced chemical vapor deposition)-grown waveguide stack consisted of 2 μm thick silicon dioxide (RI = 1.46) as bottom cladding, 7 μm thick silicon oxynitride (RI = 1.52) as core, and another 2 μm thick silicon dioxide as an upper cladding. Dielectric waveguides form an attractive solution for integrated biomedical optics^23^,^42^. Unlike polymers, dielectrics are resistant to ionic and enzymatic environments, providing negligible *in vivo* degradation^52^,^53^. In contrast to some polymer waveguides (SU-8, PDMS), they do not absorb light in the UV-blue range^54^,^55^,^56^. Since the RI of the waveguide films determines the NA of the waveguide, the PECVD processes were carefully optimized to tune the waveguide NA while maintaining film stress (72 MPa tensile for silicon oxynitride and 180 MPa compressive for silicon dioxide, respectively) and uniformity (<1%) over the entire 4-inch wafer surface. A 500 Å-thick aluminum oxide film was deposited under the waveguide films as an etch-stop, avoiding potential damage to the metal surfaces underneath. The probe shape was defined by reactive-ion etching from the front side of the wafer and then released using reactive-ion etching from the wafer backside.

ILD-GRIN jig fabrication was carried out as shown in Fig. 5e–g. The ILDs were aligned and flip-chip bonded onto the released ILD-GRIN jigs. We implemented In-Au eutectic bonding at 200 °C to achieve epi-down bonding of the ILDs on ILD-GRIN jigs. In the epi-down configuration, the diodes are flip-chip bonded with the anode facing down, so the heated active region is close to the heat sink, thereby allowing rapid heat dissipation from the active region^57^,^58^. Low-temperature indium-gold eutectic bonding was chosen since it protects the ILDs from potential thermal damage at high bonding temperatures.

**Figure 7:**
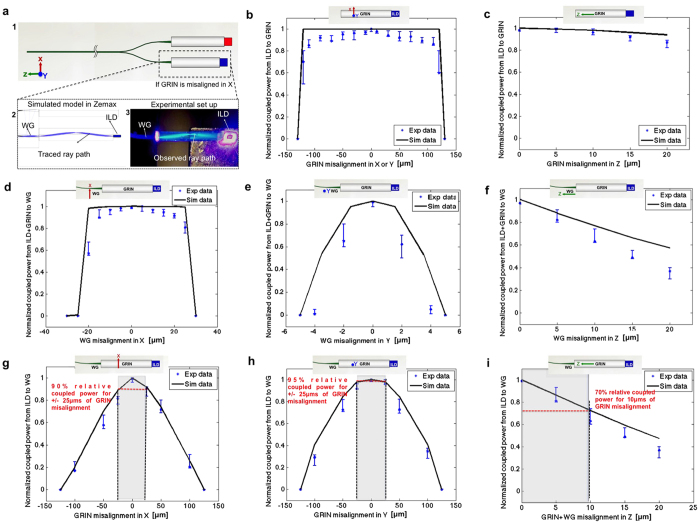
Optical system alignment tolerance analysis to evaluate assembly reproducibility and yield. (**a**) Optical model components: (1) Schematic of model components showing ILDs and GRINs coupled to the waveguide mixer (WG); (2–3) Agreement between simulated models in Zemax and experimental results obtained when GRIN lens is intentionally misaligned by 25 μm (in X-axis) while ILD and GRIN are kept stationary. The traced ray path in Zemax (2) matches very well the observed ray path in the assembled prototype device (3). (**b**,**c**) Alignment tolerance analysis for ILD-GRIN coupling when ILD is stationary but GRIN is (**b**) laterally misaligned in X or Y-axis (because GRIN lens is symmetrical about X and Y axis, misalignment in either directions leads to the same results); and (**c**) longitudinally misaligned in Z-axis. (**d**–**f**) Alignment tolerance analysis for ILD-GRIN-WG coupling when ILD and GRIN are perfectly aligned and stationary but WG is (**d**) laterally misaligned in X-axis; (**e**) laterally misaligned in Y-axis; and (**f**) longitudinally misaligned in Z-axis. (**g**–**i**) Alignment tolerance analysis for ILD-GRIN-WG coupling when ILD and WG are perfectly aligned and stationary but GRIN is (**g**) laterally misaligned in X-axis; (**h**) laterally misaligned in Y-axis; and (**i**) longitudinally misaligned in Z-axis (when GRIN displaces in Z-axis, WG displaces in Z-axis too). The inset for all graphs shows the schematic of the respective coupling interface, depicting the axis and direction of misalignment. Data points show the median of the collected data (N = 3), and error bars represent the range.

**Figure 8:**
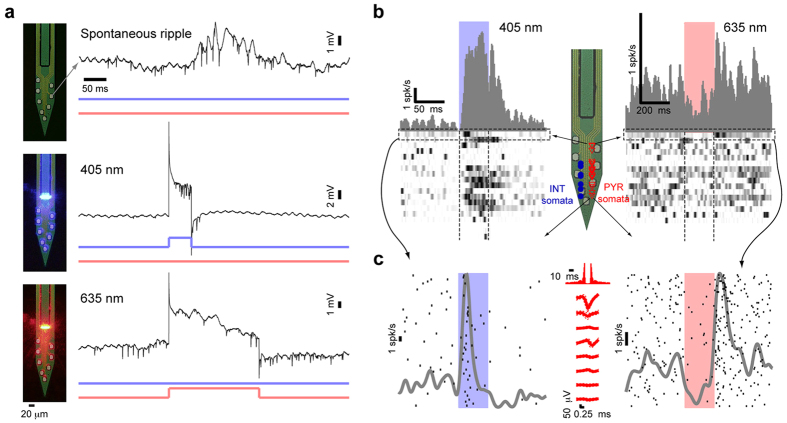
ILD-GRIN probes enable bi-directional control of pyramidal cells in the intact mouse. (**a**) Wide-band (0.3–10,000 Hz) traces recorded from CA1 pyramidal cell layer of a urethane-anesthetized mouse expressing ChR2 and eArch3 under the CaMKII promoter. **Top**, spontaneous spiking and ripple activity; **middle**, activity from the same recording site during a 100 μW pulse (power at the waveguide tip) of 405 nm light; **bottom**, recording from the same site during a 370 μW pulse of 635 nm light. Note spontaneous, induced, and silenced spiking, respectively; and stimulus-locked artifacts during ILD driving. (**b**) Spiking activity from 19 well-isolated pyramidal cells (PYR) recorded simultaneously from CA1 (same animal and session as in (**a**). Inset shows the vertical location of PYR (red triangles) and interneuron (INT, blue circles) somata relative to the probe sites. Bottom panels: heat maps showing, in each row, a peri-stimulus time histogram (PSTH) for one PYR; each PSTH was scaled to the 0–1 range. Higher rows show PSTHs for PYR with somata closer to the waveguide tip. PSTHs for simultaneously recorded INT are not shown. Most PYR (11/19; 58%) increased their spike rate (p < 0.05, Poisson test) during 405 nm light; 4/19 (21%) decreased their rate during 635 nm light. (**c**) Raster plots for a single PYR. The PYR closest to the waveguide tip exhibited consistent rate increase during 405 nm light (left) and rate decrease during 635 nm light (right; p < 0.001, Poisson test for both); each black tick indicates occurrence of one spike; different rows indicate distinct trials (light pulses). Gray curves show PSTHs (non-scaled; generated by summing spike times and convolving with a Gaussian kernel, SD = 5 ms [left] or 20 ms [right]). Plots in the center show auto-correlation histogram (top) and spike waveform (mean and SD) in the lack of any illumination. Note robust activation, suppression, and post-suppression rebound.

The optoelectrode was constructed by assembling multiple microfabricated components on a custom designed PCB (Fig. 5h). It is critical to control misalignment of optical components in all dimensions within its respective tolerance ranges (Fig. 7). Given device size, this was achieved by photolithographically-defined geometries during microfabrication, and precise assembly techniques with the aid of micromanipulators. During fabrication, all of the designed measures were achieved with ±1 μm precision. The dielectric waveguide core was made relatively tall (7 μm) to increase misalignment tolerances, and a relatively high waveguide NA (0.4228) was designed to reduce loss from angular misalignment. Assembly errors were minimized with micro-fabricated assembly jigs. The neural probe was supported on a rectangular probe jig to precisely control the vertical alignment between the probe and ILD-GRIN jig. Probe jigs were released via dicing a wafer of a given thickness (no-mask process). These jigs provide modularity in the assembly process since a given waveguide probe can be vertically aligned to any ILD height, by simply selecting a jig of a thickness matching the specific ILD-GRIN-PCB assembly. Once aligned in vertical plane, the waveguide mixer was aligned to the ILDs in the horizontal plane, and GRIN lenses were secured in their slots using an index-matching UV-curable epoxy (NOA 61, Norland Products, NJ, USA; RI = 1.56). Since the GRIN-waveguide optical junction was found more susceptible to angular misalignment errors, index-matching was not used at this junction.

The entire ILD-GRN assembly was enclosed in a micromachined light-weight polyoxymethylene cap (Fig. 2; Delrin acetal resin, McMaster-Carr, Aurora, OH, USA), designed to serve three functions: (1) block the uncoupled light escaping from optical junctions and prevent it from reaching unintended locations on brain surface; (2) facilitate convective cooling during device operation via air holes drilled in the cap; and (3) provide electrical shielding between ILDs and recording sites using a grounded 2000 Å thick gold-sputtered film. The assembled devices were wire-bonded on the PCB, which was designed to minimize capacitive coupling between the light sources and the recording traces. The PCB had 4 layers, consisting of a dedicated set of signal and ground planes for routing ILDs and recording trace connections, respectively. No overlap was made between the ILD routing and recording traces to minimize capacitive coupling. Two Omnetics connectors (A79006-001 and A790022-001, Omnetics Connector Corporation, Minneapolis, MN, USA) were used for electrical interfacing with external current sources and headstages.

### Electrophysiological procedures

All animal handling procedures were approved by the New York University Animal Care and Facilities committee, and all animal handling procedures were carried out in accordance with the approved guidelines.

Four male mice (26–42 g, 6–18 month old) were used in this study. To express ChR2 and eArch3 in pyramidal cells, two mice (B6.Cg-Tg(Camk2a-cre)T29-1Stl/J, Jackson labs #005359) were injected with a Cre-dependent virus mix (AAV5-EF1a-DIO-hChR2(H134R)-EYFP and AAV5-EF1a-DIO-eArch3.0-EYFP, University of North Carolina viral core; viral titer estimated at 4 × 10^12^ IU/ml). During virus injection, a 0.2 mm craniotomy was made at PA −1.6/ML 1.1, and 7 injections of 55 nl were made at 0.2 mm intervals from DV, 1.8 mm to 0.6 mm, to target the right dorsal hippocampus^6^. One PV::ChR2 mouse (offspring of B6;129P2-Pvalbtm2.1(cre)Arbr/J female, Jackson Labs #008069; cross-bred with B6;129S-Gt(ROSA)26Sortm32(CAG-COP4*H134R/EYFP)Hze/J male, Jackson labs #012569, Ai32) expressed ChR2 in parvalbumin-immunoreactive (PV) cells. Finally, one wild-type mouse (control; C57L/6J, Jackson Labs) was used.

Five weeks after virus injection, the animals were anesthetized (urethane, 1.5 g/kg) and prepared for acute recordings^46^. The waveguide probe was inserted at PA −1.6/ML 1.1 and gradually lowered to a depth of 600 μm. Subsequent probe movements were done in 50 or 100 μm increments over 15 min intervals until the CA1 pyramidal cell layer was approached, recognized by the appearance of multiple high-amplitude units and spontaneous ripple events^6^,^46^,^47^. Extracellular activity was filtered (0.3–10,000 Hz), amplified (400×; RHA2132, Intan), digitized (14 bit, 20 kHz digitization; KJE-1001, AmpliPex), and continuously stored on disk. For offline analysis, spike waveforms were extracted from the wide-band recorded signals and sorted into individual units^46^. Briefly, waveforms were linearly detrended, projected onto a common basis obtained by principal component analysis of the data, and sorted automatically followed by manual adjustment. Only well-isolated units (amplitude >50 μV; L-ratio <0.05; interspike interval index <0.2) were used. Subsequently each unit was tagged as excitatory/inhibitory [based on peaks/troughs in the short-time (±5 ms) pairwise cross correlation; P < 0.001, convolution test] and/or classified as putative PYR or INT (based on a Gaussian-mixture model; P < 0.05^46^). We recorded a total of 77 well isolated cells from CA1 of 4 mice in 5 sessions. Of these, 64 were PYR and 13 were INT.

Baseline neuronal activity was recorded for at least 15 minutes, followed by photostimulation (405 nm: 50 ms light pulses; 635 nm: 200 ms pulses) via a programmable DSP (25 kHz; RX8, Tucker-Davis Technologies) driving a custom-made multi-channel current source^24^. Five current levels were used per ILD, spanning the range from threshold to maximal operating level (405 nm: 15–35 mA; 635 nm: 30–45 mA). Following photostimulation, a second baseline period was recorded before the probe was moved to another target.

## Additional Information

**How to cite this article**: Kampasi, K. *et al.* Fiberless multicolor neural optoelectrode for in vivo circuit analysis. *Sci. Rep.* **6**, 30961; doi: 10.1038/srep30961 (2016).

## Supplementary Material

Supplementary Information

Supplementary movie

## Figures and Tables

**Figure 1:**
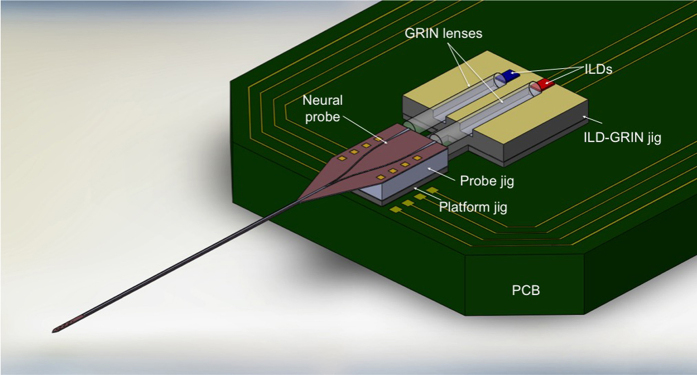
Schematic of assembled optoelectrode on a printed circuit board (PCB)^26^.

## References

[b1] Boyden E. S., Zhang F., Bamberg E., Nagel G. & Deisseroth K. Millisecond-timescale, genetically targeted optical control of neural activity. Nat. Neurosci. 8, 1263–1268 (2005). 16116447 10.1038/nn1525

[b2] Deisseroth K. Optogenetics. Nat. Methods 8, 26–29 (2011). 21191368 10.1038/nmeth.f.324 PMC6814250

[b3] Madisen L. *et al.* A toolbox of Cre-dependent optogenetic transgenic mice for light-induced activation and silencing. Nat. Neurosci. 15, 793–802 (2012). 22446880 10.1038/nn.3078 PMC3337962

[b4] Lee S.-H. *et al.* Activation of specific interneurons improves V1 feature selectivity and visual perception. Nature 488, 379–383 (2012). 22878719 10.1038/nature11312 PMC3422431

[b5] Pfeffer C. K., Xue M., He M., Huang Z. J. & Scanziani M. Inhibition of inhibition in visual cortex: the logic of connections between molecularly distinct interneurons. Nat. Neurosci. 16, 1068–1076 (2013). 23817549 10.1038/nn.3446 PMC3729586

[b6] Stark E. *et al.* Pyramidal cell-interneuron interactions underlie hippocampal ripple oscillations. Neuron 83, 467–480 (2014). 25033186 10.1016/j.neuron.2014.06.023 PMC4393648

[b7] Nagel G. *et al.* Light activation of Channelrhodopsin-2 in excitable cells of caenorhabditis elegans triggers rapid behavioral responses. Curr. Biol. 15, 2279–2284 (2005). 16360690 10.1016/j.cub.2005.11.032

[b8] Chow B. Y. *et al.* High-performance genetically targetable optical neural silencing by light-driven proton pumps. Nature 463, 98–102 (2010). 20054397 10.1038/nature08652 PMC2939492

[b9] Han X. *et al.* A high-light sensitivity optical neural silencer: development and application to optogenetic control of non-human primate cortex. Front. Syst. Neurosci. 5, 1–8 (2011). 21811444 10.3389/fnsys.2011.00018 PMC3082132

[b10] Han X. & Boyden E. S. Multilpe-color optical activation, silencing, and desynchronization of neural activity, with single-spike temporal resolution. PLoS One 2, (2007). 10.1371/journal.pone.0000299 PMC1808431 17375185

[b11] Zhang F. *et al.* Multimodal fast optical interrogation of neural circuitry. Nature 446, 633–639 (2007). 17410168 10.1038/nature05744

[b12] Gradinaru V. *et al.* Molecular and Cellular Approaches for Diversifying and Extending Optogenetics. Cell 141, 154–165 (2010). 20303157 10.1016/j.cell.2010.02.037 PMC4160532

[b13] Sohal V. S., Zhang F., Yizhar O. & Deisseroth K. Parvalbumin neurons and gamma rhythms enhance cortical circuit performance. Nature 459 698–702 (2009). 19396159 10.1038/nature07991 PMC3969859

[b14] Yizhar O. *et al.* Neocortical excitation/inhibition balance in information processing and social dysfunction. Nature 477, 171–178 (2011). 21796121 10.1038/nature10360 PMC4155501

[b15] Govorunova E. G., Sineshchekov O. A., Janz R., Liu X. & Spudich J. L. Natural light-gated anion channels: A family of microbial rhodopsins for advanced optogenetics. Science 349 (2015). 10.1126/science.aaa7484 PMC4764398 26113638

[b16] Klapoetke N. C. Independent Optical Excitation of Distinct Neural Populations. Nat Methods 11, 338–346 (2014). 24509633 10.1038/nmeth.2836 PMC3943671

[b17] Wietek J. *et al.* An improved chloride-conducting channelrhodopsin for light-induced inhibition of neuronal activity *in vivo*. Sci. Rep . 5, 14807 (2015). 26443033 10.1038/srep14807 PMC4595828

[b18] Gradinaru V. *et al.* Targeting and readout strategies for fast optical neural control *in vitro* and *in vivo*. J. Neurosci. 27, 14231–14238 (2007). 18160630 10.1523/JNEUROSCI.3578-07.2007 PMC6673457

[b19] Anikeeva P. *et al.* Optetrode: a multichannel readout for optogenetic control in freely moving mice. Nat. Neurosci. 15, 163–170 (2011). 22138641 10.1038/nn.2992 PMC4164695

[b20] Huber D. *et al.* Sparse optical microstimulation in barrel cortex drives learned behaviour in freely moving mice. Nature 451, 61–64 (2008). 18094685 10.1038/nature06445 PMC3425380

[b21] Kravitz A. V. *et al.* Regulation of parkinsonian motor behaviours by optogenetic control of basal ganglia circuitry. Nature 466, 622–626 (2010). 20613723 10.1038/nature09159 PMC3552484

[b22] Halassa M. M. *et al.* Selective optical drive of thalamic reticular nucleus generates thalamic bursts and cortical spindles. Nat. Neurosci. 14, 1118–1120 (2011). 21785436 10.1038/nn.2880 PMC4169194

[b23] Wu F. *et al.* An implantable neural probe with monolithically integrated dielectric waveguide and recording electrodes for optogenetics applications. J. Neural Eng. 10, 056012 (2013). 23985803 10.1088/1741-2560/10/5/056012 PMC4056669

[b24] Stark E., Koos T. & Buzsáki G. Diode probes for spatiotemporal optical control of multiple neurons in freely moving animals. J. Neurophysiol. 108, 349–363 (2012). 22496529 10.1152/jn.00153.2012 PMC3434617

[b25] Schwaerzle M., Seidl K., Schwarz U. T., Paul O. & Ruther P. Ultracompact optrode with integrated laser diode chips and SU-8 waveguides for optogenetic applications. In Proceedings of the IEEE International Conference on Micro Electro Mechanical Systems (MEMS) 1029–1032 (IEEE, 2013).

[b26] Kampasi K., Seymour J., Na K., Wise K. D. & Yoon E. Fiberless multicolor optoelectrodes using Injection Laser Diodes and Gradient-index lens coupled optical waveguides. In Proceedings of the 18th International Conference on Solid-State Sensors, Actuators and Microsystems (TRANSDUCERS) 273–276 (IEEE, 2015).

[b27] Riedl M. Optical Design Fundamentals for Infrared Systems Second Edition (SPIE Press, 2001).

[b28] Smith W. J. Modern Optical Engineering (Tata McGraw-Hill Education, 1966).

[b29] Hunsperger R. G. Integrated Optics (Springer-Verlag New York, 1984).

[b30] Marcatilli E. A. J. Bends in optical dielectric waveguides. Bell Syst. Tech. J. . 48, 2103–2132 (1969).

[b31] Royer S. *et al.* Multi-array silicon probes with integrated optical fibers: Light-assisted perturbation and recording of local neural circuits in the behaving animal. Eur. J. Neurosci. 31, 2279–2291 (2010). 20529127 10.1111/j.1460-9568.2010.07250.x PMC2954764

[b32] Nagel G. *et al.* Channelrhodopsin-2, a directly light-gated cation-selective membrane channel. Proc. Natl. Acad. Sci. USA. 100, 13940–13945 (2003). 14615590 10.1073/pnas.1936192100 PMC283525

[b33] Aravanis A. M. *et al.* An optical neural interface: *in vivo* control of rodent motor cortex with integrated fiberoptic and optogenetic technology. J. Neural Eng. 4, S143–S156 (2007). 17873414 10.1088/1741-2560/4/3/S02

[b34] Mattis J. *et al.* Principles for applying optogenetic tools derived from direct comparative analysis of microbial opsins. Nat. Methods. 9, 159–172 (2011). 22179551 10.1038/nmeth.1808 PMC4165888

[b35] Elwassif M. M., Kong Q., Vazquez M. & Bikson M. Bio-heat transfer model of deep brain stimulation induced temperature changes. J. Neural Eng. 3, 306 (2006). 17124335 10.1088/1741-2560/3/4/008

[b36] Shen K. fei & Schwartzkroin P. A. Effects of temperature alterations on population and cellular activities in hippocampal slices from mature and immature rabbit. Brain Res. 475, 305–316 (1988). 3214738 10.1016/0006-8993(88)90619-1

[b37] Andersen P. & Moser E. I. Brain temperature and hippocampal function. Hippocampus 5, 491–498 (1995). 8646277 10.1002/hipo.450050602

[b38] Long M. A. & Fee M. S. Using temperature to analyse temporal dynamics in the songbird motor pathway. Nature 456, 189–194 (2008). 19005546 10.1038/nature07448 PMC2723166

[b39] Wu F. *et al.* Monolithically integrated μLEDs on silicon neural probes for high-resolution optogenetic studies in behaving animals. Neuron 88, 1136–1148 (2015). 26627311 10.1016/j.neuron.2015.10.032 PMC4702503

[b40] Abdelkader H. I., Hausien H. H. & Martin J. D. Temperature rise and thermal rise-time measurements of a semiconductor laser diode. Rev. Sci. Instrum. . 63, 2004–2007 (1992).

[b41] Wise K. D. Silicon microsystems for neuroscience and neural prostheses. IEEE Eng. Med. Biol. Mag. 24, 22–29 (2005). 16248114 10.1109/memb.2005.1511497

[b42] Zorzos A. N., Boyden E. S. & Fonstad C. G. Multiwaveguide implantable probe for light delivery to sets of distributed brain targets. Opt. Lett. 35, 4133–4135 (2010). 21165114 10.1364/OL.35.004133 PMC3010404

[b43] Buzsáki G., Horváth Z., Urioste R., Hetke J. & Wise K. High-frequency network oscillation in the hippocampus. Science 256, 1025–1027 (1992). 1589772 10.1126/science.1589772

[b44] Chuong A. S. *et al.* Noninvasive optical inhibition with a red-shifted microbial rhodopsin. Nat. Neurosci. 17, 1123–1129 (2014). 24997763 10.1038/nn.3752 PMC4184214

[b45] Han X. *et al.* Millisecond-timescale optical control of neural dynamics in the nonhuman primate brain. Neuron 62, 191–198 (2009). 19409264 10.1016/j.neuron.2009.03.011 PMC2830644

[b46] Stark E. *et al.* Inhibition-Induced theta resonance in cortical circuits. Neuron 80, 1263–1276 (2013). 24314731 10.1016/j.neuron.2013.09.033 PMC3857586

[b47] Stark E., Roux L., Eichler R. & Buzsáki G. Local generation of multineuronal spike sequences in the hippocampal CA1 region. Proc. Natl. Acad. Sci. USA. 112, 10521–10526 (2015). 26240336 10.1073/pnas.1508785112 PMC4547251

[b48] Biran R., Martin D. C. & Tresco P. A. Neuronal cell loss accompanies the brain tissue response to chronically implanted silicon microelectrode arrays. Exp. Neurol. 195, 115–126 (2005). 16045910 10.1016/j.expneurol.2005.04.020

[b49] Polikov V. S., Tresco P. A. & Reichert W. M. Response of brain tissue to chronically implanted neural electrodes. J. Neurosci. Methods. 148, 1–18 (2005). 16198003 10.1016/j.jneumeth.2005.08.015

[b50] Szarowski D. H. *et al.* Brain responses to micro-machined silicon devices. Brain Res. 983, 23–35 (2003). 12914963 10.1016/s0006-8993(03)03023-3

[b51] Blanche T. J., Spacek M. A., Hetke J. F. & Swindale N. V. Polytrodes: High-Density Silicon Electrode Arrays for Large-Scale Multiunit Recording. J. Neurophysiol. 93, 2987–3000 (2005). 15548620 10.1152/jn.01023.2004

[b52] Worhoff K., Hilderink L. T. H., Driessen A. & Lambeck P. V. Silicon oxynitride - A versatile material for integrated optics applications. J. Electrochem. Soc. 149, F85–F91 (2002).

[b53] Wörhoff K., Klein E., Hussein G. & Driessen A. Silicon oxynitride based photonics. In Proceedings of 2008 10th Anniversary International Conference on Transparent Optical Networks, ICTON. 3, 266–269 (IEEE, 2008).

[b54] Rubehn B., Wolff S. B. E., Tovote P., Lüthi A. & Stieglitz T. A polymer-based neural microimplant for optogenetic applications: design and first *in vivo* study. Lab Chip 13, 579–588 (2013). 23306183 10.1039/c2lc40874k

[b55] Kwon K. & Li W. Integrated multi-LED array with three-dimensional polymer waveguide for optogenetics. In Proceedings of the IEEE International Conference on Micro Electro Mechanical Systems (MEMS) 1017–1020 (2013).

[b56] Im M., Cho I. J., Wu F., Wise K. D. & Yoon E. A dual-shank neural probe integrated with double waveguides on each shank for optogenetic applications. Proc. Annu. Int. Conf. IEEE Eng. Med. Biol. Soc. 5480–5483 (2011). 10.1109/IEMBS.2011.6091398 22255578

[b57] Liu X. *et al.* Comparison between epi-down and epi-up bonded high-power single-mode 980-nm semiconductor lasers. IEEE Trans. Adv. Packag. 27, 640–646 (2004).

[b58] Jin T. & Ronnie W. Semiconductor Laser Diode Technology and Applications (InTech, 2012).

