# Supplementary material for: Fiberless multicolor neural optoelectrode for in vivo circuit analysis
Source: Sci Rep. 2016 Aug 3;6:30961. doi: 10.1038/srep30961 (PMC4971539; doi:10.1038/srep30961)
Supplement: Supplementary Information [file srep30961-s1.pdf]

Supplementary Information

Title:

Fiberless multicolor neural optoelectrode for in vivo circuit analysis

Author list:

Komal Kampasi[1], Eran Stark[3,4,5], John Seymour[2], Kyounghwan Na[2], Herbert G. Winful[2], György Buzsáki[3], Kensall D. Wise[1,2], Euisik Yoon[1,2]

Author affiliation:

[1]Department of Biomedical Engineering, University of Michigan, Ann Arbor, MI 48105, USA

[2]Department of Electrical Engineering and Computer Science, University of Michigan, Ann Arbor, MI 48105, USA

[3]NYU Neuroscience Institute, School of Medicine, East River Science Park, Alexandria Center, 450 East 29th St, 9th Floor, New York, NY 10016, USA

[4]Department of Physiology and Pharmacology, Sackler Faculty of Medicine, Tel Aviv University, 69978 Tel Aviv, Israel

[5]Sagol School of Neuroscience, Tel Aviv University, 69978 Tel Aviv, Israel

*E-mail: esyoon@umich.edu, Phone: (734) 615-4469, Fax: (734) 763-9324.

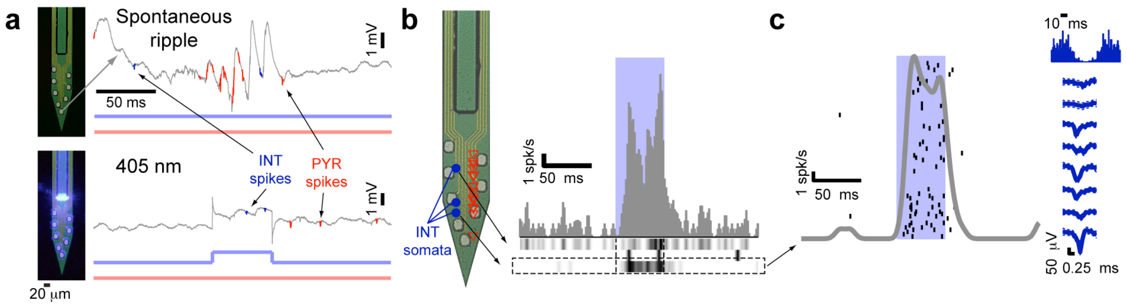

Supplementary Figure S1: ILD-GRIN probes mediate interneuron activation in the intact mouse (in attachments)

(**a**) Wide-band (0.3-10,000 Hz) traces recorded from CA1 pyramidal cell layer of a urethane-anesthetized mouse expressing ChR2 under the PV promoter. Top panel shows spontaneous spiking and ripple activity. Bottom panel shows activity from the same recording site during a 50 µW pulse (power at the waveguide tip; driving current, 25 mA) of 405 nm light. Note induced INT spikes (blue), suppressed PYR spiking (red), and stimulus-locked artifacts during the light pulse.

(**b**) Spiking activity from 3 well-isolated INT recorded simultaneously from CA1 (same animal and session as in (**a**)). Left panel shows the vertical location of the PYR (red triangles) and INT (blue circles) somata relative to the probe sites. Heat maps (bottom right) show, in each row, a PSTH for one INT, scaled to the 0-1 range. Higher rows show PSTHs for INT with somata closer to the waveguide tip. PSTHs for simultaneously recorded PYR are not shown. All three recorded INT increased their spike rate ($p<0.05$, Poisson test) during violet light pulses.

(**c**) Raster plots for a single INT, exhibiting a 6.8-fold rate increase during 405 nm light pulses ($p<0.001$, Poisson test). Each black tick marks the occurrence of one spike. Gray curve shows PSTH (non-scaled; generated by summing spike times and convolving with a Gaussian kernel, SD=5 ms). Plots at right show the auto-correlation histogram (top) and spike waveform (mean and SD) in the lack of any illumination. Note robust activation during violet light pulses.

Supplementary Movie S1: Multicolor light at the output of a waveguide port (30 µm x 7 µm) of ILD-GRIN coupled optoelectrode (in attachments).

The video demonstrates intensity-controlled, independent activation of different wavelengths for an ILD-GRIN-coupled optoelectrode. The video begins with independent activation of 405 nm wavelength, then

shows independent activation of 635 nm wavelength and finally shows simultaneous emission of both 405 nm and 635 nm wavelengths (to give a color-mixed output) at the same waveguide port.
